# CircRNA accumulation in the aging mouse brain

**DOI:** 10.1038/srep38907

**Published:** 2016-12-13

**Authors:** Hannah Gruner, Mariela Cortés-López, Daphne A. Cooper, Matthew Bauer, Pedro Miura

**Affiliations:** 1 University of Nevada, Reno, Department of Biology, 1664 N. Virginia St, Reno, NV, 89557, USA; 2 Centro de Ciencias Genómicas UNAM Cuernavaca, Av. Universidad S/N Col. Chamilpa, 62210, Cuernavaca, Morelos, Mexico; 3 Davidson Academy, 1164 N. Virginia St, Reno, NV, 89503, USA

## Abstract

Circular RNAs (circRNAs) are a newly appreciated class of RNAs expressed across diverse phyla. These enigmatic transcripts are most commonly generated by back-splicing events from exons of protein-coding genes. This results in highly stable RNAs due to the lack of free 5′ and 3′ ends. CircRNAs are enriched in neural tissues, suggesting that they might have neural functions. Here, we sought to determine whether circRNA accumulation occurs during aging in mice. Total RNA-seq profiling of young (1 month old) and aged (22 month old) cortex, hippocampus and heart samples was performed. This led to the confident detection of 6,791 distinct circRNAs across these samples, including 675 novel circRNAs. Analysis uncovered a strong bias for circRNA upregulation during aging in neural tissues. These age-accumulation trends were verified for individual circRNAs by RT-qPCR and Northern analysis. In contrast, comparison of aged versus young hearts failed to reveal a global trend for circRNA upregulation. Age-accumulation of circRNAs in brain tissues was found to be largely independent from linear RNA expression of host genes. These findings suggest that circRNAs might play biological roles relevant to the aging nervous system.

Circular RNAs (circRNAs) are a large class of non-coding molecules observed in a diverse range of life forms that include archea^1^, plants^2^, and mammals including human^3^,^4^. Eukaryotic circRNAs are most commonly formed by back-splicing events from exons of protein-coding genes where a downstream 5′ splice site joins with an upstream 3′ splice site^3^,^5^. In *Drosophila*, circRNAs were found to be most highly and specifically expressed in the brain compared to non-neural tissues^6^. This trend was extended to mouse and human, showing that preferential circRNA expression in the brain is conserved among species^3^,^7^,^8^. Certain circRNAs are expressed in a specific spatial and temporal pattern in the brain^7^,^9^. Interestingly, circRNAs generated from genes required for synaptic functions are enriched in synaptoneurosomes and neuropil samples, some of which were found to be upregulated in response to altered neuronal activity^10^. This neural propensity for circRNAs hints that there might be functional roles for these molecules in neurons.

Thus far, the biological functions of circRNAs remain largely mysterious. One proposed function for circRNAs is to sequester or “sponge” microRNAs, preventing them from acting on target mRNA 3′ UTRs. The mammalian circRNA *CDR1as (ciRS-7*) was found to possess an impressive 74 miR-7 binding sites which effectively sponged miR-7 in zebrafish brains when ectopically expressed, causing a defect in midbrain development^3^,^11^. Other circRNAs have also been proposed to sponge microRNAs^11^,^12^,^13^. However, recent genome-wide analysis argues against a broad microRNA sponging function for most circRNAs^14^. Another proposed function of circRNAs is related to their biogenesis during splicing. Back-splicing events prevent the production of linear transcript from the same pre-mRNA^15^. The competition between back-splicing and linear splicing might act as a general mechanism to regulate mRNA processing from shared host-genes. Some circRNAs have been found to tether proteins together, influencing protein-protein interactions and protein subcellular localization^16^,^17^. Cellular functions for circRNAs in diverse processes are just starting to be explored, including potential roles in cancer^18^ and cardiac function^12^. Although these are encouraging examples of the diverse actions of circRNAs, the functional roles of the thousands of circRNAs in brain tissues^7^ (with the notable exception of *CDR1as*) remain unknown.

Recent evidence suggests circRNAs might have roles in the aging brain. Out of 2,513 circRNAs identified in *Drosophila*, 262 were significantly upregulated >2-fold in 20 day old heads compared to 1 day old heads^6^. A unique feature of circRNAs is their lack of 5′ and 3′ ends, which enhances their stability compared to their mRNA counterparts^15^,^19^, which might contribute to their age-accumulation. The enhanced stability of circRNAs is a possible reason these molecules display increased abundance in non-proliferating cells^7^,^20^. Alterations of splicing patterns with age have also been observed in various organisms and tissues^21^,^22^,^23^,^24^,^25^, which might also affect circRNA levels. It is unknown whether circRNAs also accumulate during aging in the brains of other animals.

To investigate the relationship between circRNAs and aging in mammalian tissues, we performed ribo-depleted total RNA-seq to detect circRNAs in cortex, hippocampus and heart of young (1 month old) and aged (22 month old) C57BL/6 mice. We uncovered a genome-wide trend for increased circRNA expression in brain tissues of old versus young mice. Interestingly, this trend was not observed in heart. CircRNA expression patterns were extensively validated by RT-qPCR and Northern analysis. The increased abundance of circRNAs during aging was found to be largely independent of gene expression from their host genes. Consistent with this finding, profiling of linear RNAs in brain tissues did not reveal a global upregulation trend. Together, these results suggest that increased abundance of circRNAs might impact age-related decline in neural function.

## Results

### Mapping circRNAs from total RNA-seq data of young and aged mouse tissues

**Figure 1:**
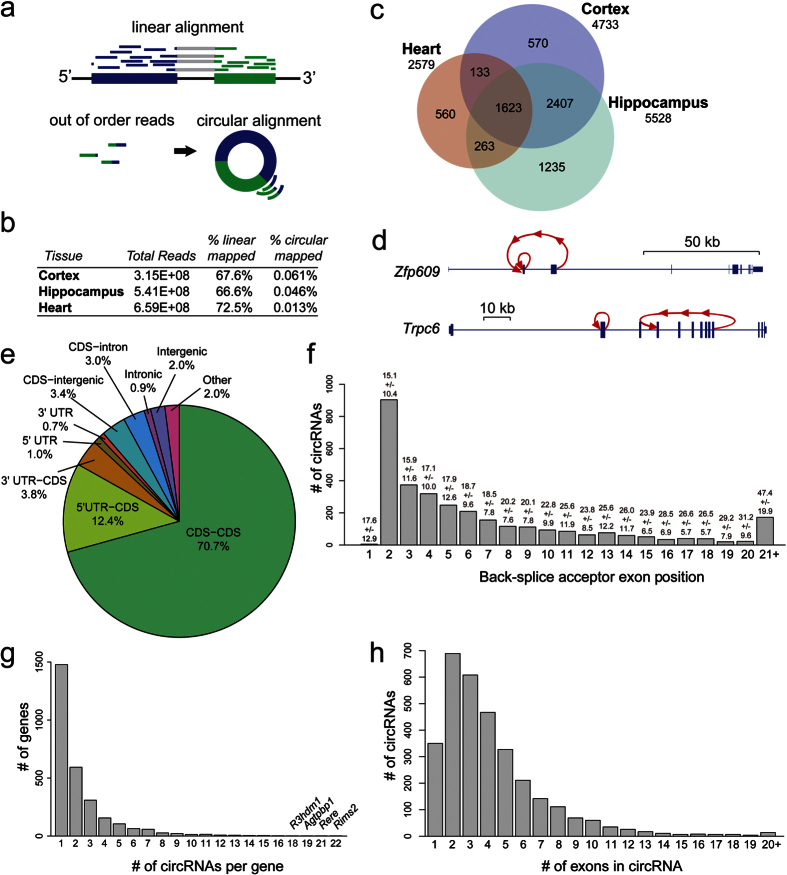
Mapping and genomic features of circRNAs. **(a)** Linear alignment of RNA-seq reads contrasted to mapping split “out of order” reads to back-spliced exons. **(b)** Read mapping to linear RNAs and circRNAs using 6 libraries each from cortex, hippocampus and heart. **(c)** Venn diagram showing overlap of annotated circRNAs among tissues. **(d)** Schematic of circularized exons (red) from *Zfp609* and *Trpc6* genes. **(e)** Distribution of circRNAs in the genome. **(f)** Distribution of circRNAs with respect to most upstream circularized exon. On top of each bar the average number of exons within the host gene for the circRNAs in the group is listed+/− standard deviation. **(g)** Distribution of # of circRNAs per gene. **(h)** Distribution of # of exons found within circRNAs contained within known genes (intergenic circRNAs are excluded).

Thousands of circRNAs have been recently annotated and surveyed across different mouse tissues and cell lines^3^,^7^,^10^. To investigate the global levels of circRNAs in the aging mouse nervous system, we profiled circRNAs in cortex, hippocampus and heart from 1 month old mice (1 mo) and 22 month old (22 mo) mice. Ribo-depleted total RNA-seq libraries were prepared and sequenced with paired-end 125 nt reads. All conditions were sequenced in biological triplicates. We categorized 1 mo as the young time point because previous work profiling circRNAs in the brain from embryonic day 18 through 1 mo of age uncovered a trend for circRNA upregulation^10^. Most circRNAs are of low abundance and only reads that map to back-spliced junctions can be used for quantification (Fig. 1a). Thus, we decided to sequence at a very high depth and generated in total 1.52 billion sequence reads from 18 different libraries. We first mapped reads to previously identified circRNAs^3^,^7^,^26^. For each circRNA, a scaffold for alignment was created with 100 nt of sequence on either side of the back-spliced junction. After alignment, reads that were likely PCR duplicates were removed using Picard (http://broadinstitute.github.io/picard/). We set a minimum cutoff of 6 reads across the 6 libraries for each tissue (a minimum average of 1 read per biological replicate), which is more stringent than previous annotations that required a minimum of 2 unique reads across a back-spliced junction^3^,^7^. Using these criteria, we annotated 6,116 previously identified circRNAs in our datasets.

In addition to mapping to known circRNAs, we attempted to identify novel circRNAs using a previously described circRNA identification algorithm, find_circ^3^. This algorithm uses reads that do not align in a linear fashion to a reference genome as input, then examines these reads for splice donor and acceptor sites that are out-of-order relative to the genome. After applying find_circ to our data, we annotated 675 novel circRNAs.

In total, after filtering the data for multi-gene spanning circRNAs (see “Experimental validation of aging circRNA expression patterns”), we annotated 6,791 unique circRNAs. The annotation pipeline is summarized in Supplementary Fig. S1. The majority of reads mapped linearly to the genome, and less than 0.1% of reads generated mapped unambiguously to circRNAs (Fig. 1b). Full statistics of read alignment to circRNA and linear RNAs are found in Supplementary Table S1. Approximately double the number of circRNAs were found in brain tissues compared to heart tissue (Fig. 1c), which is consistent with previous findings^10^. The majority of annotated circRNAs in cortex were also found in hippocampus, and vice versa (Fig. 1c). Of the 6,791 circRNAs, 1,623 (24%) were expressed in all three tissues (Fig. 1c). A recent annotation using find_circ reported >14,000 circRNAs to be expressed in mice with 24.2% of annotated circRNAs having only 2 unique reads supporting their expression from multiple libraries^7^. Our annotation set is more conservative in that it is based on having a 6 unique read minimum. We next set out to determine the genomic features of these annotated circRNAs.

### Genomic features and diversity of circRNAs

The vast majority of circRNAs (6,664/6,791) were generated from annotated protein-coding genes. Examples of typical circRNAs generated from the *Zfp609* and *Trpc6* genes are shown in Fig. 1d. The detected circRNAs emanated from a variety of genomic regions, including untranslated regions (UTRs), intergenic regions, but most commonly protein-coding exons (CDS-CDS) (70.7%), followed by exons spanning coding and 5′ UTR regions (5′ UTR-CDS) (12.4%) (Fig. 1e). In agreement with previous findings^4^,^6^, the splice accepting circularized exon was most commonly exon 2, and we observed a general preference for circRNAs to emanate from exons at the 5′ ends of genes (Fig. 1f). Although it was most common for circRNA producing genes to generate a single circRNA, there were 1466 genes that generated two or more circRNAs. For example, the *Rere* and *Rims2* genes each generated 21 and 22 circRNAs, respectively (Fig. 1g). Finally, there was a great diversity in the number of exons contained within circRNAs. Of the 6,791 circRNAs, 5,275 contained between 1–5 exons and 1,496 had 6 or more exons (Fig. 1h).

### Brain expressed circRNAs display an upregulation trend with aging

**Figure 2:**
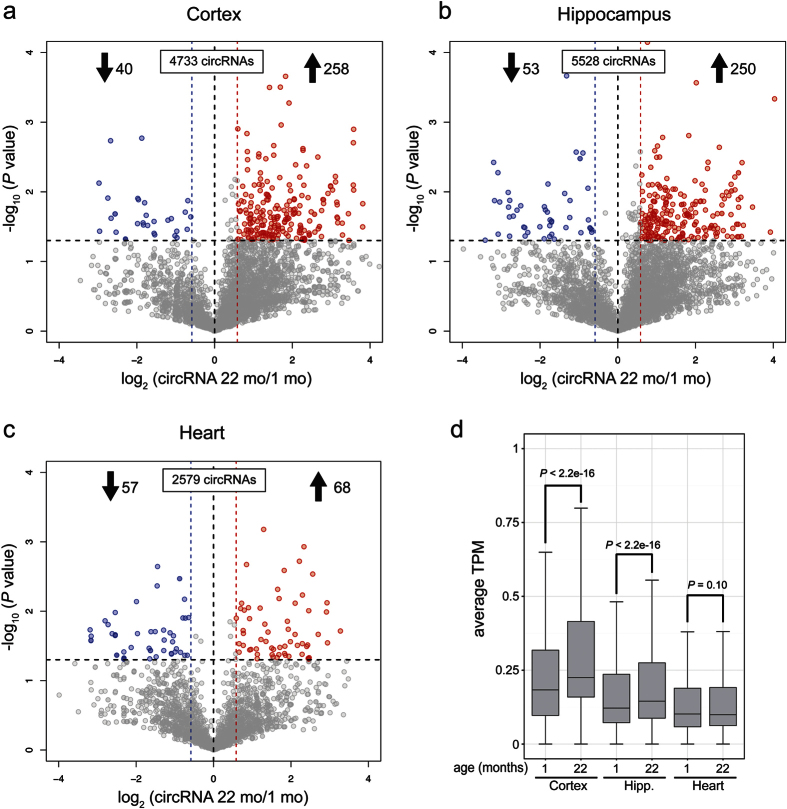
Differential circRNA expression between young (1 mo) and old (22 mo) tissues. Volcano plots showing −log_10_ (*P* value) versus log_2_ fold difference in circRNA abundance in transcripts per million reads (TPM) between 22 mo and 1 mo tissues. circRNAs included in the analysis are those with 6 or more combined back-spliced reads in a given tissue. Red circles denote significant age-upregulated circRNAs whereas blue circles denote significant age-downregulated circRNAs (*P* < 0.05). Fold-change cutoff is set at 1.5. **(a)** Cortex circRNAs. More than 6-fold circRNAs are upregulated versus downregulated with age. Note the density of gray circles (non-significant changes) in the bottom right quadrant, indicating greater expression in 22 mo versus 1 mo. **(b)** Hippocampus circRNAs. Nearly 5-fold more circRNAs are upregulated versus downregulated with age. **(c)** Heart circRNAs. Note the reduced number of annotated circRNAs compared to brain regions and lack of age-upregulation trend. **(d)** Boxplots comparing circRNAs as measured by TPM among the three tissues. Hipp, Hippocampus. *P* value represents Wilcoxon rank sum test with continuity correction. n = 3 biological replicates for each tissue and age.

We next performed expression profiling to identify circRNAs that were differentially expressed during aging. CircRNA abundance was quantified as circular Transcripts Per Million Reads (TPM), and differential expression between samples was calculated. We set a minimum expression fold change cutoff of 1.5, and compared 22 mo versus 1 mo samples for each tissue. Volcano plots revealed a striking trend of circRNA age upregulation in cortex and hippocampus samples, but not in heart (Fig. 2a–c). Read counts, normalized values, TPM counts, fold changes and *P* values for all the libraries are found in Supplementary Tables S2–S4. In cortex samples we detected 4,733 circRNAs in total (≥6 reads/6 libraries). We found that 258 (5.4%) circRNAs were significantly upregulated >1.5-fold and 40 (0.8%) were significantly downregulated >1.5-fold with age (Fig. 2a). In hippocampus, of the 5,528 circRNAs, 250 (4.5%) were significantly upregulated >1.5-fold during aging, whereas 53 (1.0%) were downregulated >1.5-fold (Fig. 2b). In addition to the significant changes (red and blue circles in Fig. 2a–c), a strong trend for upregulation that did not reach significance (Fig. 2a,b- gray circles in bottom right quadrant) was evident. Thus, our reported number of upregulated circRNAs might be an underestimation of the age-regulated circRNA population.

In stark contrast to the neural tissues, a bias for increased circRNA abundance in aged samples was not found in heart. In total there were 68 circRNAs expressed significantly higher in 22 mo versus 1 mo hearts (2.7% of total), and 57 circRNAs expressed at significantly lower levels (2.2%) (Fig. 2c). This suggests that the global trend for circRNA upregulation during aging might be neural-specific.

In addition to reporting individual circRNAs regulated by age, we compared the global levels of circRNAs between the ages for each tissue. Wilcoxon rank sum test with continuity correction revealed highly significant changes for old versus young cortex and hippocampus circRNA TPM values (*P* < 2.2 E-16) (Fig. 2d). In contrast, no significant change was found between old versus young heart (*P* = 0.10) (Fig. 2d). Thus, circRNAs are globally upregulated during aging in brain tissues.

### Experimental validation of aging circRNA expression patterns

**Figure 3:**
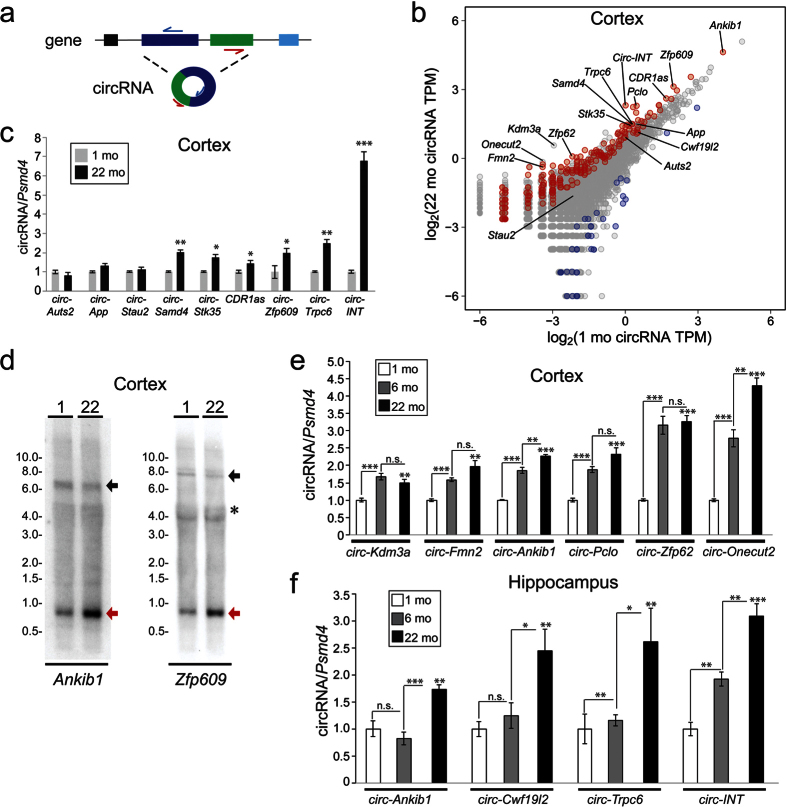
Validation of circRNA abundance changes during aging. **(a)** Schematic of RT-qPCR strategy to detect circRNAs using outward facing primers. **(b)** Scatterplot generated from cortex circRNA data (same values as in Fig. 2a). Red circles represent significantly upregulated circRNAs (22 mo versus 1 mo samples) and blue circles represent significantly downregulated circRNAs (*P* < 0.05). Select circRNAs validated by RT-qPCR (panels c,e,f) are noted. **(c)** RT-qPCR validation of circRNA expression changes in cortex (n = 3). **(d)** Northern blots performed on 22 mo and 1 mo cortex terminator exoribonuclease treated RNA show age-upregulation of circRNAs from the *Ankib1* and *Zfp609* genes. Red arrow denotes circRNA. Black arrow denotes mRNA. *, denotes background hybridization to 28S rRNA. **(e)** Expression changes for select circRNAs using RT-qPCR among 1 mo, 6 mo, and 22 mo samples (n = 4). **(f)** Expression changes for select circRNAs among 1 mo, 6 mo, and 22 mo hippocampus samples. Asterisk on 22 mo bars reflect significant changes versus 1 mo (n = 4). Error bars represent standard error of the mean. n.s., not significant. *P < 0.05; **P < 0.01, ***P < 0.001.

We performed RT-qPCR experiments to validate the differential expression trends. For quantifying circRNAs by qPCR, we employed outward facing primers that permit the exclusive detection of circularized exons (Fig. 3a). Experimentally validated circRNAs are highlighted in a scatterplot comparing old and young cortex (Fig. 3b). Increased expression between 22 mo versus 1 mo cortex samples was confirmed for *circ-Stk35* (mm9_circ_002813), *CDR1as, circ-Zfp609* (mm9_circ_004501), and *circ-Trpc6* (mm9_circ_013636) (Fig. 3c). The highest fold change we confirmed (~7-fold by qPCR) was a circle from an intergenic region which we called *circ-INT* (mm9_circ_017175). The fold changes detected by RNA-seq (Supplementary Table S2) were in good agreement with the RT-qPCR quantifications (Fig. 3c). We also confirmed the trends of several circRNAs that were not significantly changed between 22 mo and 1 mo samples according to the RNA-seq data. These included *circ-Auts2* (mm9_circ_005046), *circ-App* (mm9_circ_016270) and *circ-Stau2* (mmu_circ_0008346). As expected, all three did not show a statistically different change by RT-qPCR (Fig. 3c). Due to the low number of reads for certain circRNAs, it is possible that many upregulation trends exist that were not quantifiable at the employed depth of sequencing. For instance, a 2.9-fold increase of *circ-Samd4* (mm9_circ_005305) determined from the RNA-seq data was not statistically significant (*P* = 0.083) (Supplementary Table S2). However, RT-qPCR indicated a significant ~2-fold upregulation of *circ-Samd4* in the aged cortex sample (Fig. 3c). This supports our assertion that the differential expression analysis is likely an underestimate of the total compendium of age-upregulated circRNAs. Expression trends were also confirmed by Northern analysis using a probe that detects both linear and circular RNA forms. CircRNAs from the *Ankib1* gene (mm9_circ_000903) and *Zfp609* gene (mm9_circ_004501) were increased in 22 mo vs 1 mo cortex samples (Fig. 3d). In contrast, levels of mRNA from these genes visualized on the same blots were not increased with age.

Given the strong upregulation trends for circRNAs in 22 mo compared to 1 mo brain tissues, we wanted to determine if this age-accumulation was progressive. We thus performed additional RT-qPCR validations from cortex using an intermediate age of 6 mo. The expression of 6/6 circRNAs tested were found to be significantly increased at 6 mo compared to 1 mo. Of these, 2/6 circRNAs were also significantly increased between 6 mo to 22 mo (Fig. 3e). It is also of note that both *circ-Kdm3a* (mmu_circ_0013507) and *circ-Onecut2* (mmu_circ_0007448) had *P* values of >0.05 in the RNA-seq data (1 mo versus 22 mo), and thus are not part of the 258 cortex age-upregulated circRNAs. Again, this emphasizes that the RNA-seq analysis of circRNA differential expression likely underestimates the global cohort of age-upregulated circRNAs.

We performed additional validations of circRNA expression trends among the three aging timepoints in the hippocampus. We found 2/4 circRNAs to be significantly increased between 1 mo and 6 mo, and 4/4 circRNAs were significantly increased between 6 mo and 22 mo (Fig. 3f). From profiling this limited set of circRNAs, it appears that age-accumulation of circRNAs is progressive in brain tissues, and is not restricted to early or late phases of aging. This is in agreement with the progressive accumulation of circRNAs observed during *Drosophila* aging^6^. The PCR products resulting from these amplifications were run in agarose gels to determine if a single PCR product was generated. For all cases, a single band was detected (Supplementary Fig. S2).

**Figure 4:**
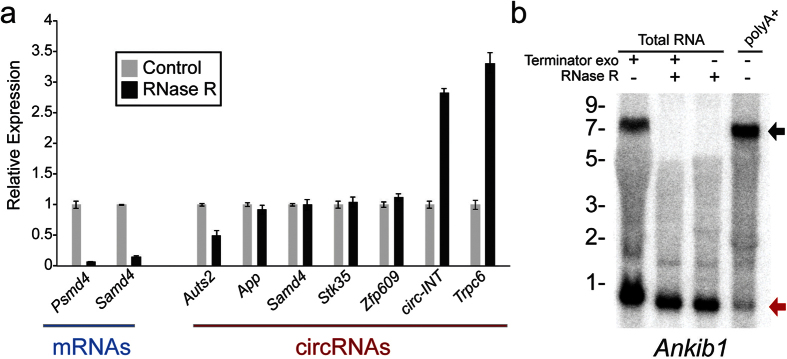
RNase R validation of circRNA annotations. **(a)** RT-qPCR experiments show that 7 detected circRNAs are resistant to RNase R treatment. Input RNA was a mixed sample of 7 week old and 12 month old cortex. **(b)** Northern blot using a probe to detect circular (red arrow) and linear (black arrow) products from the *Ankib1* gene in cortex. The circRNA band is not degraded by RNase R treatment, whereas the mRNA band is eliminated. Also note the reduced detection of circRNA species in polyA+ RNA samples. Terminator 5′-Dependent Exoribonuclease treatment was employed to remove ribosomal RNA which can interfere with target detection when performing glyoxal Northern blots.

Resistance to RNase R, a 3′ to 5′ exoribonuclease, provides additional support that a predicted RNA is a bonafide circRNA^3^,^6^,^19^. We treated cortex total RNA with RNase R, and then performed cDNA synthesis and qPCR. All 6/6 circRNAs tested were more resistant to RNase R than the linear mRNA controls, of which 2/2 were highly degraded by RNase R (Fig. 4a). Further confirmation of individual circRNAs was obtained by Northern blot analysis. As predicted, we detected circular and linear products from the *Ankib1* gene in total RNA samples treated with Terminator 5′-Phosphate Dependent exoribonuclease to remove ribosomal RNA. Treatment with RNase R completely ablated the *Ankib1* mRNA band, while the circRNA band was unaffected (Fig. 4b). In addition, the *Ankib1* band was severely diminished in polyA+ selected RNA, further supporting the circular nature of this transcript (Fig. 4b). Together, this evidence provides strong support for *circ-Ankib1* as a bonafide circRNA.

Experimental validation of individual circRNA trends also led to improvements in our annotation pipeline. From our pipeline, we identified 87 loci that spanned multiple genes. Previous work has annotated 31 and 46 of these loci as circRNAs^7^,^3^. We examined these putative circRNAs more closely and observed that in many cases these “multi-gene” circRNAs spanned exons of neighbouring genes with highly similar sequences (e.g. *C4a/C4b*). To test the validity of these multi-gene circRNAs we characterized one of the most highly expressed multi-gene circRNAs, *C4a/C4b*. We performed PCR on cortex cDNA followed by Sanger sequencing to determine whether the multi-gene spanning *C4a/C4b* circRNA annotation could be validated. The sequenced product was found to be consistent with linear-spliced exons and not back-spliced exons within the *C4b* gene (Supplementary Fig. S3). In contrast, we were able to confirm back-spliced exons for all single-gene spanning circRNAs we tested (11/11 circRNAs), including: *circ-Zfp609, circ-Trpc6, circ-Stk35, circ-INT, circ-Ankib1, circ-Onecut, circ-Cwf19l2, circ-Kdm3a, circ-Pclo, circ-Fmn2*, and *circ-Zfp62* (Supplementary Table S5). Thus, multi-gene spanning circRNAs were filtered out of the annotation pipeline (Supplementary Fig. S1).

### Classification of genes generating neural age-upregulated circRNAs

**Figure 5:**
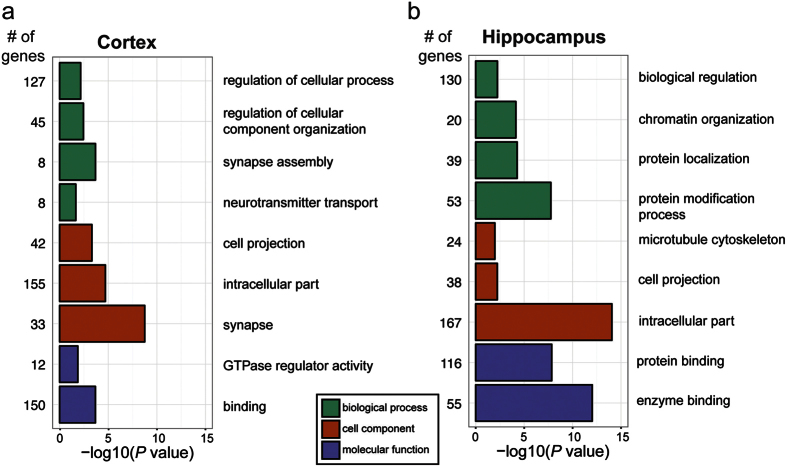
Age-upregulated circRNA host gene GO analysis. Gene ontology (GO) analysis was performed on host genes of significantly upregulated circRNAs in **(a)** cortex and **(b)** hippocampus. Notable categories from the analysis are shown (see Supplemental Tables S6 & S7 for full lists). Enriched terms are grouped by GO category- Biological Process (green), Cell Component (orange), and Molecular Function (purple).

We performed Gene Ontology (GO) analysis on the host genes that produce the age-regulated circRNAs from the different tissues (Supplementary Tables S6–S8). Although GO categories are assumed to reflect functions of proteins derived from a given gene, it is possible that loss of function studies contributing to these annotations might also have disrupted the circRNA loci. Moreover, changes in back-splicing to generate circRNAs could impact protein expression from a gene. For cortex age-upregulated circRNAs, GO analysis revealed enrichment in cellular component categories of “synapse” (*P* = 1.77E-09), and Biological Process category “synapse assembly” (*P* = 2.07E-04) (Fig. 5a). These categories included circRNAs emanating from the *Piccolo (Pclo)* and *Erc2* genes, which are both involved in synaptic vesicle fusion^27^,^28^. *Circ-Rims2* is another highly expressed and age-upregulated circRNA that has been previously validated and found to be preferentially expressed in synaptoneurosomes^7^; the protein product of *Rims2* is involved in synaptic vesicle priming^29^. No enrichment of GO terms was found for the age-downregulated circRNAs in cortex.

In contrast to the cortex, enrichment in GO terms related to the synapse was not uncovered in the hippocampus. Enrichment for Biological Process terms related to protein modification, including “protein modification process” (*P* = 1.76E-08) and “chromatin modification” (*P* = 7.28E-05) (Supplementary Table S7) were uncovered for age-upregulated circRNAs in the hippocampus (Fig. 5b). For the molecular function category there was enrichment for “protein binding” (*P* = 1.42E-08), as was found for cortex upregulated circRNAs, and “enzyme binding” (*P* = 9.36E-13). Included in this list were circRNAs from genes with notable functions. For instance, several genes involved in transcription regulation, such as *Hdac4, NFATc3, Top1*, and *Mtf2* generated circRNAs that were upregulated (Supplementary Table S3). We also identified age-upregulated hippocampus circRNAs that arose from genes with pertinent neural functions (*Rims1, Rims2, Grik4*), and identified a circRNA produced from the *Rtn3* gene, a negative regulator of the enzyme that cleaves amyloid precursor protein^30^. As found for the cortex, there was no enrichment of GO terms for downregulated circRNAs in the hippocampus (Supplementary Table S3). For heart circRNAs, no notable enriched terms were found for age-upregulated or downregulated circRNAs (Supplementary Table S8). These results suggest brain-expressed age-upregulated circRNAs might have biological functions.

### MicroRNA targeting to brain expressed circRNAs

The only characterized biological function for a neural circRNA is activity as a microRNA sponge when exogenously expressed^3^. We searched the loci of expressed cortex and hippocampus circRNAs for conserved microRNA target sites using TargetScan^31^. For the input set of microRNAs, we selected only those classified by TargetScan as broadly conserved (92 microRNAs). We restricted our analysis to microRNA seed regions that were conserved among mouse, human and rhesus monkey. This strict conservation threshold was applied since most circRNAs are generated from protein-coding exons, which have high conservation compared to 3′ UTRs. As most circRNAs have introns spliced out, we filtered the target sites so that only those overlapping previously annotated exons were reported. Most circRNAs in cortex (Supplementary Table S9) and hippocampus (Supplementary Table S12) were found to harbor conserved microRNA target sites.

We wanted to determine whether age-upregulated circRNAs tended to have a greater number of target sites for particular microRNAs. This would be consistent with the hypothesis that particular circRNAs might be upregulated during aging to inhibit or “sponge” particular microRNAs. In Supplementary Tables S9–S14 each circRNA loci is listed with the 5 most common microRNA target sites within their exons, and the respective number of instances of these microRNA target sites. Lists are also provided for age-upregulated (Supplementary Tables S10 and S13) and downregulated circRNAs (Supplementary Tables S11 and S14).

There were several age-upregulated circRNAs that harbored a high incidence of target sites for microRNAs with known neural functions. For instance, *CDR1as*, the miR-7 sponging circRNA^3^,^11^, harbored 36 highly conserved miR-7 target sites in our analysis and was confirmed to be upregulated during aging in the cortex (Fig. 3c & Supplementary Table S10). Three target sites for miR-7 were also found in exons of a circRNA from the *Hecw1* locus (mmu_circ_0004501), which was upregulated in both old cortex and hippocampus (Supplementary Tables S10 and S13). A circRNA from the *Zfyve9* gene (mm9_circ_014815) that was age-upregulated in hippocampus harbored several different microRNA target sites: 3 target sites for miR-9, a microRNA with roles in neural development and neural pathologies^32^, 1 target site for miR-124, a highly abundant brain miRNA that is implicated in central nervous system disorders^33^, and 1 miR-7 target site (Supplementary Table S13). In addition, a cortex age-upregulated circRNA from the *Acin1* gene (mmu_circ_0005278) harbored 4 target sites for miR-9, (Supplementary Tables S10 and S13, Supplementary Fig. S4). It is possible that a functional role for such circRNAs is to provide increased sponging of neural microRNAs during aging; however, we did not uncover an trend for age-upregulated circRNAs versus all expressed circRNAs to harbor more neural microRNA target sites. This argues against microRNA sponging serving as a general function of age-accumulated circRNAs.

### Linear RNAs in brain regions lack an age-upregulation trend

**Figure 6:**
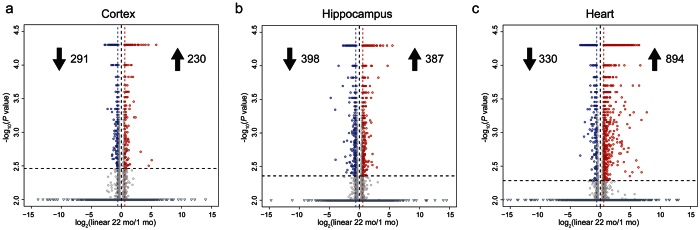
Linear RNA expression changes between young (1 mo) and old (22 mo) mice. Volcano plots showing significant differences in FPKM values for linear RNA abundance between 1 mo and 22 mo tissues. The number of upregulated and downregulated linear RNAs are shown. Red circles denote significant age-upregulated linear RNAs whereas blue circles denote age-downregulated linear RNAs (*P* < 0.05). Values on the Y-axis (−log_10_(*P* value)) less than 2.0 are represented as triangles. Fold change cutoff was set at 1.5. **(a)** Young versus old cortex. **(b)** Young versus old hippocampus. **(c)** Young versus old heart.

We next sought to determine how the upregulation of circRNAs in brain tissues during aging compared to expression changes of linear RNAs. We mapped and quantified linear RNAs corresponding to GENCODE annotations between young and old tissues using the Tuxedo suite of RNA-seq analysis tools^34^. Volcano plots showing expression differences of linear RNAs for cortex, hippocampus and heart are found in Fig. 6. In contrast to the circRNA results, there was no general trend for upregulation of linear RNAs in the cortex and hippocampus.

For cortex samples, using a fold change cutoff of 1.5 we found 230 linear RNAs were upregulated whereas 291 were downregulated (Fig. 6a). GO analysis for age-upregulated and age-downregulated linear RNAs in cortex is found in Supplementary Table S18. In agreement with previous aging studies^24^,^35^, we found that *C4b*, a component of the complement system, and the long noncoding RNA *Neat1*, a core component of the nuclear paraspeckle suborganelle, were highly upregulated by 7-fold and 5-fold, respectively.

In the hippocampus, 387 linear RNAs were upregulated whereas 398 were downregulated (Fig. 6b). In contrast to cortex, enrichment of several notable categories were found for the age-upregulated hippocampus linear RNAs. High enrichment was found for “defense response” (*P* = 5.40E-07) and “immune response” (*P* = 1.54E-07) (Supplementary Table S19). Also of interest was the enrichment for the Biological Process category of “RNA binding” (*P* = 3.79E-06). Age downregulated linear RNAs had highly significant enrichment for “nervous system development” (*P* = 2.10E-13). In aged heart tissue we did find a bias for upregulation of linear RNAs. We identified 894 significantly increased genes and 330 significantly decreased genes when comparing old and young hearts (Fig. 6c). Similar to hippocampus, we found in heart that age-upregulated linear RNAs were enriched for terms related to the immune system such as “defense response” (*P* = 2.31E-30) (Supplementary Table S20).

**Figure 7:**
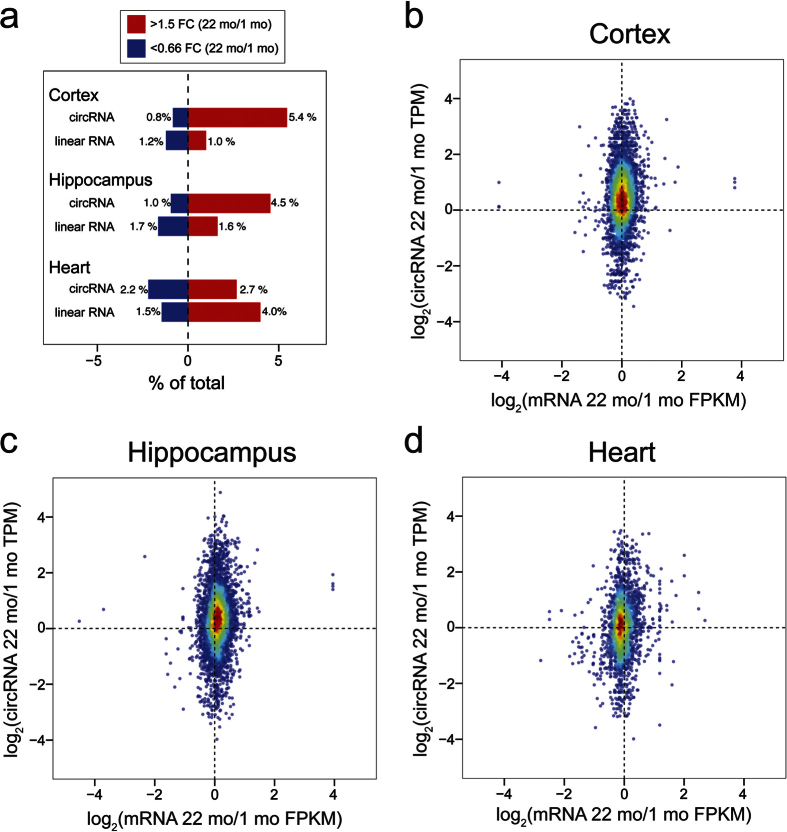
CircRNA increases during aging are largely independent of host gene expression. **(a)** Barplots comparing the percentage of RNAs differentially expressed between 22 mo versus 1 mo samples from each tissue. FC = Fold Change. For cortex and hippocampus, the trend for age-upregulation is much stronger globally for circRNAs compared to linear RNAs. In contrast, a greater upregulation trend is seen for linear RNAs compared to circRNAs in heart. **(b)** Density plots comparing the log_2_ fold changes in circRNA transcripts per million (TPM) in 22 mo versus 1 mo samples on the Y-axis and the corresponding fold change FPKM of linear RNAs (22 mo versus 1 mo) from the corresponding gene on the X-axis. Cortex circRNA/mRNA density plot shows a strong trend for circRNA accumulation independent of cognate linear RNA expression from the same gene. **(c)** Hippocampus circRNA versus mRNA density plot shows similar trends as cortex. **(d)** Heart circRNA/mRNA density plot lacks circRNA age-upregulation trend.

Thus, as a class of RNAs, circRNAs are more positively correlated with aging compared to linear RNAs in brain tissues. Linear RNAs in cortex and hippocampus did not show an aging bias, whereas >4-fold more circRNAs were upregulated versus downregulated in old versus young brain tissues (Fig. 7a). Principal component analysis was performed on the linear RNA data (Supplementary Fig. S5a) and circRNA data (Supplementary Fig. S5b). This analysis effectively distinguished the different tissues with close clustering of the replicates. The neural tissues, but not heart, could be distinguished by age using either the linear RNA or circRNA data. Thus, to predict tissue source or age, it appears that global circRNA profiles might be equally predictive as global linear RNA profiles.

### Aging circRNA expression trends are mostly independent of host gene expression

As most circRNAs emanate from protein-coding genes, it is possible that some circRNA abundance changes could reflect general transcriptional output of the host gene. We cross-referenced the genes from the linear RNA analysis that were upregulated >1.5-fold with circRNA loci that were upregulated >1.5-fold during aging. Minimal overlap was uncovered, suggesting that the circRNA abundance changes are largely independent of the general transcription from their host genes. Only 17/258 age-upregulated cortex circRNAs had linear RNA expression concomitantly increased. Similarly, we found only 20/250 age-upregulated hippocampus circRNAs that also had their linear RNA expression concomitantly increased. To address the global independence of circRNA regulation in aging compared to mRNA changes from the same host gene, we generated density plots comparing circRNA TPM counts (22 mo/1 mo) against the Fragments Per Kilobase of transcript per Million mapped reads (FPKM) of linear RNA expression from the same host gene (22 mo/1 mo). For cortex and hippocampus, an upward shift along the y-axis was clearly visible in these plots, whereas no bias was found on the x-axis (Fig. 7b,c). Thus, circRNA upregulation in cortex and hippocampus on a genome-wide scale is independent of cognate mRNA expression. As expected, a shift along the y-axis was not found for heart, indicating that there was no change in the global expression of circRNAs versus linear RNAs during aging (Fig. 7d). The independence of circRNA expression and linear RNA expression from the same host gene suggests that factors and/or cellular conditions that influence the stability or biogenesis of circRNAs might underlie their accumulation during aging.

## Discussion

Here, we provide the first demonstration of an age-accumulation trend for circRNAs in mammals. Using exceptionally deep total RNA-seq data, we uncovered a global bias for circRNA accumulation in aging mouse cortex and hippocampus, but not in the heart (Fig. 2). The circRNA expression increases were largely independent of mRNA changes from their host genes (Fig. 7). These observations in mice, combined with previous findings in *Drosophila*^6^, might indicate that age-accumulation of circRNAs is a universal feature of brain tissues.

We found that ~5% of circRNAs were significantly increased in brain tissues of old versus young mice, whereas only ~1% were decreased (Fig. 2a,b). Although there were many shared age-upregulated circRNAs between cortex and hippocampus, there were also many age-upregulated circRNAs that were unique to each tissue. This was reflected in contrasting enrichments in GO terms for the two tissues (Fig. 5). This could point to brain-region specific roles for age-accumulated circRNAs or might reflect cellular differences between the tissues during aging. For example, genes producing circRNAs that were upregulated during aging in the cortex were enriched for synaptic GO terms, but this trend was not found for hippocampus (Fig. 5). Perhaps differences in the types of synapses lost during aging between these tissues might explain the discrepancy^36^. Many circRNAs are quantified with a low number of reads which can preclude the detection of significant differences between conditions. It is possible that low expression levels of particular circRNAs in either cortex or hippocampus might have resulted in the detection of age-accumulation trends in one tissue and not the other. Alternative methods targeted to particular circRNAs (e.g. custom microarrays or hybridization-based selection prior to RNA-seq) might prove to be more accurate for low abundance circRNA differential expression analysis.

We found that a greater proportion of circRNAs detected were upregulated in aged brain tissues compared to the proportion of linear RNAs (mostly comprised of mRNAs). This raises the possibility that circRNAs might serve as useful aging biomarkers, although the utility of this approach might be limited, as it could require brain tissue biopsies. There have been many previous studies that employed microarrays or RNA-seq to profile aging tissues. A meta-analysis of aging transcriptome changes in various organisms and tissues revealed a slight bias toward upregulation versus downregulation^37^. Only a handful of linear RNAs are consistently upregulated across multiple tissues, species and studies^37^,^38^. Some of these RNAs include long non-coding RNAs such as *Neat1* and protein-coding mRNAs such as *C4b* and *ApoD*, which we also found to be upregulated in our study. Along these lines, total RNA-seq profiling uncovered a bias for upregulation of non-coding RNAs but not mRNAs in 28 mo versus 12 mo rats^39^. One main motivation for profiling aging transcriptomes is to uncover factors that might play a role in the aging process in hopes that therapeutics might be designed to counteract age-related decline or to enhance lifespan. It is an open question whether upregulation of circRNAs and other non-coding RNAs in the brain is a consequence of aging or might contribute to age-related decline.

What factors contribute to the age-accumulation of circRNAs? Given that most of the age-accumulation events were independent of host-gene expression of linear RNAs (Fig. 7), and that a bias for linear RNA increase during aging in brain tissues was not found (Fig. 6), it is unlikely that enhanced gene transcription during aging is involved. We hypothesize that two mechanisms might contribute- (1) enhanced stability of circRNAs, and (2) alterations in alternative splicing.

CircRNAs are exceptionally stable^19^ and thus might progressively accumulate over time as a result of ongoing transcription, whereas mRNAs are more rapidly degraded. In tissues with a high degree of proliferative cells, the accumulation of circRNAs due to their stability is predicted to be less than tissues such as the brain that have a large proportion of post-mitotic cells. This is simply because when cells die, circRNAs are lost. In support of this hypothesis, circRNAs were found to be less abundant in highly proliferative cells and cancer cells^40^ and reduced circRNA levels were found in brain samples of glioma patients versus healthy patient brain samples^41^. Moreover, it was found that the dramatic induction of circRNAs during P19 cell neural differentiation is decreased in proportion to the amount of GFAP positive cells (proliferative glial cells) in culture^7^.

It is unclear why a bias for circRNA upregulation during aging was found in brain tissues and not heart (Fig. 2). In rhesus macaque, RNA-seq profiling of circRNAs during aging was performed in skeletal muscle, and similarly did not uncover an age-upregulation trend^42^. Perhaps muscle cells in general do not accumulate circRNAs with aging. Although cardiomyocytes might be slightly more proliferative than neurons^43^, it seems unlikely that the lack of an age-upregulation trend in the heart could be solely attributed to differences in proliferative status. Multiple studies have shown that brain tissues express more circRNAs than non-neural tissues^3^,^7^,^8^,^10^, and circRNAs are abundantly increased in various models of neural differentiation^7^. Perhaps neural-specific or neural-enriched factors that enhance circRNA stability influence circRNA abundance in neurons and age-accumulation. There are many cell types found in the brain, including neurons, endothelial cells, astrocytes, microglia, and oligodendrocytes, which have been found to have distinct gene expression profiles^44^. Fluorescence-activated cell sorting of these different cell types from the brains of aging mice followed by total RNA-seq could clarify the relative contribution of these cell types to circRNA age-upregulation.

Another line of evidence supporting a mechanism of enhanced stability is that circRNA accumulation during aging begins early, and is progressive. In *Drosophila*, circRNA accumulation was found to occur between 1 and 5 days of age^6^. Similarly, circRNAs from the mouse brain are globally increased between embryonic day 18 and 1 month of age^10^. Although we did not perform RNA-seq on an intermediate time point between 1 mo and 22 mo in this study, we did obtain RT-qPCR evidence for a progressive accumulation of multiple circRNAs at 6 mo in aging cortex and hippocampus (Fig. 3e,f). This suggests that circRNA accumulation during aging is a continuous process, and not particularly characteristic of extremely old age.

In addition to the enhanced stability of circRNAs playing a role for age-accumulation, regulation of alternative splicing might be important. Many groups have found that age alters global splicing patterns in human and mouse^21^,^22^,^23^,^24^,^25^. Thus, it is plausible that age-related increases in back-splicing might be responsible for some of the age-related circRNA accumulation trends. The splicing factors Muscleblind and Quaking have been found to positively influence circRNA biogenesis^15^,^20^, whereas several hnRNP and SR proteins have been found to suppress circRNA biogenesis^45^. Although we did not find correlations of these factors in our aging RNA-seq datasets to support a role in the age-accumulation of circRNAs, we did find a GO enrichment of “RNA-binding” for our hippocampus age-upregulated mRNAs. This enrichment, however, was not found in the cortex data. Perhaps other aspects governing neural splicing factor activity with age, such as post-translational modification status, could underlie trends of circRNA accumulation and enhanced alternative splicing with age.

The accumulation of circRNAs in the aging brain might be protective, detrimental, or simply be an innocuous correlate. Some mRNAs known to be overexpressed with age are thought to be beneficial; for instance, *ApoD* and *Mgst1* are upregulated during age^37^ and have known roles in protecting against oxidative stress^46^,^47^. Uncovering functions of age-upregulated circRNAs is of particular importance given that old age is highly predictive of neurodegenerative diseases such as Parkinson’s and Alzheimer’s disease^48^,^49^. Many splicing changes that occur in the brains of patients with neurodegenerative diseases are shared with non-affected old individuals^23^. Profiling of circRNAs in various neurodegenerative models to identify common upregulated circRNAs will be useful to determine which circRNAs warrant further investigation in functional studies. It might be the case that individual circRNAs could impact neuronal function during aging, either contributing to deterioration, or providing a protective affect. Alternatively, an influence of circRNAs on the aging brain might result from the cumulative increase of hundreds of circRNAs in the cell.

Moving forward, it will be important to identify whether age-accumulation of circRNAs occurs in the human brain. A common RNA signature of aging between mouse and human is lacking^38^, which raises questions concerning the relevance of mouse aging RNA profiling experiments to understanding human aging. Age-upregulated circRNAs that are conserved between human and mouse would thus be ideal candidates for future studies of circRNA function.

## Methods

### Animal care

All mouse experiments were approved by the University of Nevada, Reno IACUC and were in accordance with NIH guidelines. For old mice, C57Bl/6 mice were obtained from the National Institutes of Aging (NIA) aged rodent colony. Young (1 mo) and 6 mo C57Bl/6 mice were obtained from Charles River Laboratories.

### RNA processing

After sacrificing mice by CO_2_ asphyxiation, tissues were dissected and flash frozen in liquid nitrogen. Tissues were pulverized on dry ice using a mortar and pestle, and RNA was extracted using the Universal RNeasy Mini kit (Qiagen). RNA quality was assessed by Bioanalyzer (Agilent) and quantified using PicoGreen (ThermoFisher Scientific) at the Nevada Genomics Center.

### Library preparation and high-throughput sequencing

Libraries were prepared using the Illumina TruSeq Stranded Total RNA Library Prep Kit. RNA fragmentation was performed as recommended by the manufacturer for the cortex libraries (94 °C × 8 min). For hippocampus and heart libraries, modified conditions were used to increase size of the cloned fragments (85 °C × 5 min). Prepared libraries were sequenced at New York Genome Center (New York, NY) using a HiSeq 2500 to obtain paired-end 125 bp reads. All samples for each aged condition were sequenced in biological triplicate.

### Accession numbers

Raw fastq files from the RNA-seq data are deposited at NCBI Sequence Read Archive and accession numbers are listed in Supplementary Table S1.

### Experimental Validation of circRNAs

To confirm individual circRNAs, RNA was reverse transcribed using random hexamers and Superscript III (Invitrogen). PCR products were gel extracted and Sanger sequenced (Nevada Genomics Center), or first cloned into PCR 2.1- TOPO TA vector (Invitrogen) prior to Sanger sequencing. qPCR was performed on a BioRad CFX96 real time PCR machine using SYBR select mastermix for CFX (Applied Biosystems). The delta delta Ct method was used for quantification using CFX manager software. Experiments were performed using technical quadruplicates. Student’s t-test was used to test for statistical significance. RNaseR (Epicentre) treatment was performed for 10 minutes at 37 °C (2.5 units/μg RNA). To stop the reaction, RNA was extracted using acid phenol chloroform.

For Northern analysis, polyA+ RNA was obtained from total RNA using NucleoTrap mRNA kit (Machery-Nagel). In order to reduce ribosomal RNA, 100 μg total RNA was treated with 12 units of Terminator Exonuclease (Epicentre) for one hour at 30 °C. Glyoxal-DMSO was used to denature RNA and electrophoresis was performed using 1% agarose gels in BPTE. Downward capillary transfer to a Nytran membrane in 20X SSC was performed using Turboblotter kit (Whatman). After transfer and washing, UV crosslinking was performed using Stratalinker (Stratagene). Probing was performed using ULTRAhyb hybridization buffer (Ambion) and α 32-P dCTP-labeled (Perkin Elmer) double stranded DNA probes prepared with the Megaprime DNA labeling system (GE Healthcare). The DNA probes consisted of PCR products amplified from cortex cDNA. Hybridization was carried out overnight at 50 °C. After washes, blots were exposed to phosphorscreen and images were acquired using a Typhoon 7000IP phosphorimager (GE Healthcare).

### CircRNA prediction and mapping

find_circ (https://github.com/marvin-jens/find_circ) (v1.2 default parameters, output filtered for a minimum of 6 reads) was used to obtain a set of predicted circRNA loci from the RNA-seq datasets. We also obtained previous circRNA annotations from mouse brain tissues and cell lines^3^,^7^. A custom circRNA reference scaffold was generated that included the *de novo* and previously published circRNA annotations. The reference sequences consisted of 100 nt before and after the back-spliced junction. This permitted 200 nt circRNA references to map the 125 nt reads. The reference sequences were retrieved using Bedtools getfasta^50^ and merged using custom scripts. Bowtie2 (parameters: –very-sensitive –score-min = C,−15,0)^51^ was used for mapping the individual mate reads to the junctions of the circRNA reference scaffold. After mapping, Picard (https://broadinstitute.github.io/picard/) (parameters: MarkDuplicates ASSUME_SORTED = true REMOVE_DUPLICATES = true) was used to remove duplicates from our mapping data. We removed 77 loci that spanned multiple genes, as these were likely mapping errors (see results). To quantify the number of mapped reads for each junction we used featureCounts^52^ (parameters: -C -t exon).

### Normalization and cutoffs

After duplicate removal, a cutoff of 6 reads across the 6 libraries for each tissue (3 biological replicates per condition) was set. This led to 6,791 unique circRNA annotations. To account for difference in library depth among samples, scaling by circRNA Transcripts Per Million of reads (TPM) in each library was performed. Fold change of TPM values were generated between 22 mo and 1 mo. A cutoff of 1.5-fold change difference was used. T-test (*P* < 0.05) was used across the normalized TPM values to identify differentially expressed circRNAs. Correction for multiple hypothesis testing was not performed.

### Mapping and quantification of linear RNA expression

We aligned the reads to the NCBI37/mm9 reference genome (custom parameters) and used TopHat^34^ to align to GENCODE annotations (M1 release, NCBIM37, Ensembl 65). For differential expression analysis, Cuffdiff was performed using Benjamini-Hochberg correction for multiple-testing with a cutoff of 1.5-fold change to consider a linear RNA as differentially expressed.

### Circular to linear ratio comparison

In order to normalize expression of circRNAs to linear RNAs from the same host gene, FPKM values from the Cuffdiff output for each host gene were used. Calculation of significant fold change differences for circRNA TPM/host gene FPKM was performed using t-test (*P* < 0.05).

### Gene Ontology analysis

Gene ontology (GO) analysis was performed using the online enrichment tool from GO database powered by PANTHER classification system (http://pantherdb.org/). Lists of genes corresponding to linear RNAs or circRNA parental genes were provided as input for PANTHER significant enrichment detection tool. Each gene in the list was compared against the complete GO annotation database released on 05-20-2016. The option of Bonferroni correction for multiple hypothesis testing was used for all cases. Molecular Function, Biological Process and Cellular Component were analyzed. The cutoff for detecting significant enrichment was set to *P* < 0.05.

### PCA analysis

Principal component analysis was performed in R (R Base Package) using as input the log_10_ normalized expression values (FPKMs for linear RNAs and TPMs for circRNAs) adding a pseudo count of 1 to avoid nulls. Plots were generated with ggplot2 (http://ggplot2.org).

### Plots

Genome-wide analysis plots were generated in R. Volcano plots were generated using R base functions, scatterplots were generated using ggplot2, and density plots were generated using the LSD package (https://cran.r-project.org/web/packages/LSD/).

### MicroRNA target site analysis

MicroRNA target sites in exons of circRNA loci were identified using TargetScan 7.0 Perl script^31^. The TargetScan collection of broadly conserved mouse microRNAs (92 microRNAs) were used to identify corresponding target sites. Conservation of microRNA seed sequences among mouse, human, and rhesus monkey in the circRNA loci was required. We restricted analysis of microRNA target sites to exons of the circRNA loci by filtering for exons corresponding to GENCODE annotation M1 release.

## Additional Information

**How to cite this article**: Gruner, H. *et al*. CircRNA accumulation in the aging mouse brain. *Sci. Rep.* **6**, 38907; doi: 10.1038/srep38907 (2016).

**Publisher's note:** Springer Nature remains neutral with regard to jurisdictional claims in published maps and institutional affiliations.

## Supplementary Material

Supplementary Material

Supplementary Table S1

Supplementary Tables S2–S4

Supplementary Table S5

Supplementary Table S6–S8

Supplementary Tables S9–S14

Supplementary Tables S15–S17

Supplementary Tables S18–20
