# Supplementary material for: CircRNA accumulation in the aging mouse brain
Source: Sci Rep. 2016 Dec 13;6:38907. doi: 10.1038/srep38907 (PMC5153657; doi:10.1038/srep38907)
Supplement: Supplementary Material [file srep38907-s1.pdf]

SUPPLEMENTARY MATERIAL

CircRNA accumulation in the aging mouse brain

Hannah Gruner, Mariela Cortés-López, Daphne A. Cooper, Matthew Bauer and Pedro Miura

Supplementary Figure Legends:

Figure S1: **Pipeline for circRNA annotation.** Using previously published annotations combined with de novo circRNA annotation using find_circ, 6,791 circRNAs were annotated from 18 total RNA-seq libraries.

Figure S2: PCR products from RT-qPCR reactions used to detect circRNAs. (a) RT-qPCR products of circRNAs tested for age accumulation in 1 mo vs 22 mo cortex (See **Fig. 3C**). Products were amplified from mixed 1 and 22 mo cortex cDNA and were found to migrate at the expected sizes. **(b)** CircRNA products tested for expression in 1 mo, 6 mo, and 22 mo cortex and hippocampus (See **Fig. 3e,f**). RT-qPCR products from 22 mo cortex cDNA are shown, except for *Cwf19l2* which was amplified from 22 mo hippocampus cDNA.

Figure S3: Multi-gene spanning circRNAs are likely artifacts. (a) Example of previously annotated multi-gene circRNA (mm9_circ_012873) predicted to be formed from the paralogs *C4b* and *C4a*. **(b)** Schematic of strategy to test for the presence of *C4b*/*C4a* circRNA using Sanger sequencing. The adjacent mRNA-specific exon (blue) downstream of the predicted circularized *C4b* exon (green) is only one nucleotide different than the back-spliced exon from the *C4a* gene (orange). **(c)** Sanger sequencing performed on cortex RT-PCR products generated from outward facing primers reveals that the sequenced product matched the predicted mRNA sequence, but not the circRNA sequence. **(d)** RNase R treatment followed by qRT-PCR reveals that mm9_circ_012873 multi-gene spanning circRNA is not RNase R resistant, and thus the

product is likely indicative of a linear RNA. Amplification of the cDNA from *Psmd4* linear RNA was performed as a positive control.

Figure S4: MicroRNA seed sites in *Acin1* circRNA (mmu_circ_0005278)**.** The circRNA mmu_circ_0005278 is upregulated during aging in cortex and harbors 4 target sites for miR-9, a neural-specific microRNA (highlighted in pink). Screen shot from UCSC genome browser tracks shows broadly conserved microRNA target sites at the *Acin1* locus (yellow shaded box). Sequence alignment from multiple vertebrate species is shown at one of the miR-9 target sites. Matching of miR-9-5p to one target site is shown (7mer-m8 match). See Supplementary Tables S9-S14 for microRNA target site analysis. See Supplementary Table S2 for circRNA expression changes during aging in cortex.

Figure S5: **Principal component analysis on linear and circular RNAs. (a)** PCA performed on linear RNA data expressed in FPKM. Neural versus cardiac tissues are separated by PC1. The neural tissues (cortex and hippocampus) are separated from each other by PC3. Separation by age (1 mo vs 22 mo) along PC3 is visible for the neural tissues but not the heart. **(b)** PCA performed on circRNA data expressed in TPM. Separation by tissue type (neural vs cardiac- PC1) and specific tissues (cortex versus hippocampus versus heart- PC3) is observed. Separation by age (1 mo vs 22 mo- PC3) is found for cortex and hippocampus, but not heart.

Supplementary Table Legends:

Table S1: RNA-seq read statistics

Table S2: Cortex circRNA expression data

Table S3: Hippocampus circRNA expression data

Table S4: Heart circRNA expression data

Table S5: Oligonucleotides used for PCR, qPCR and Northern blot probes

Table S6: Gene Ontology analysis for circRNA age-regulated circRNA loci in cortex

Table S7: Gene Ontology analysis for circRNA age-regulated circRNA loci in hippocampus

Table S8: Gene Ontology analysis for circRNA age-regulated circRNA loci in heart

Table S9: MicroRNA target site analysis for all cortex circRNAs

Table S10: MicroRNA target site analysis for age-upregulated cortex circRNAs

Table S11: MicroRNA target site analysis for age-downregulated cortex circRNAs

Table S12: MicroRNA target site analysis for all hippocampus circRNAs

Table S13: MicroRNA target site analysis for age-upregulated hippocampus circRNAs

Table S14: MicroRNA target site analysis for age-downregulated hippocampus circRNAs

Table S15: Linear RNA differential expression analysis for young versus old cortex

Table S16: Linear RNA differential expression analysis for young versus old hippocampus

Table S17: Linear RNA differential expression analysis for young versus old heart

Table S18: Gene Ontology analysis for age-regulated linear RNA loci from cortex

Table S19: Gene Ontology analysis for age-regulated linear RNA loci from hippocampus

Table S20: Gene Ontology analysis for age-regulated linear RNA loci from heart

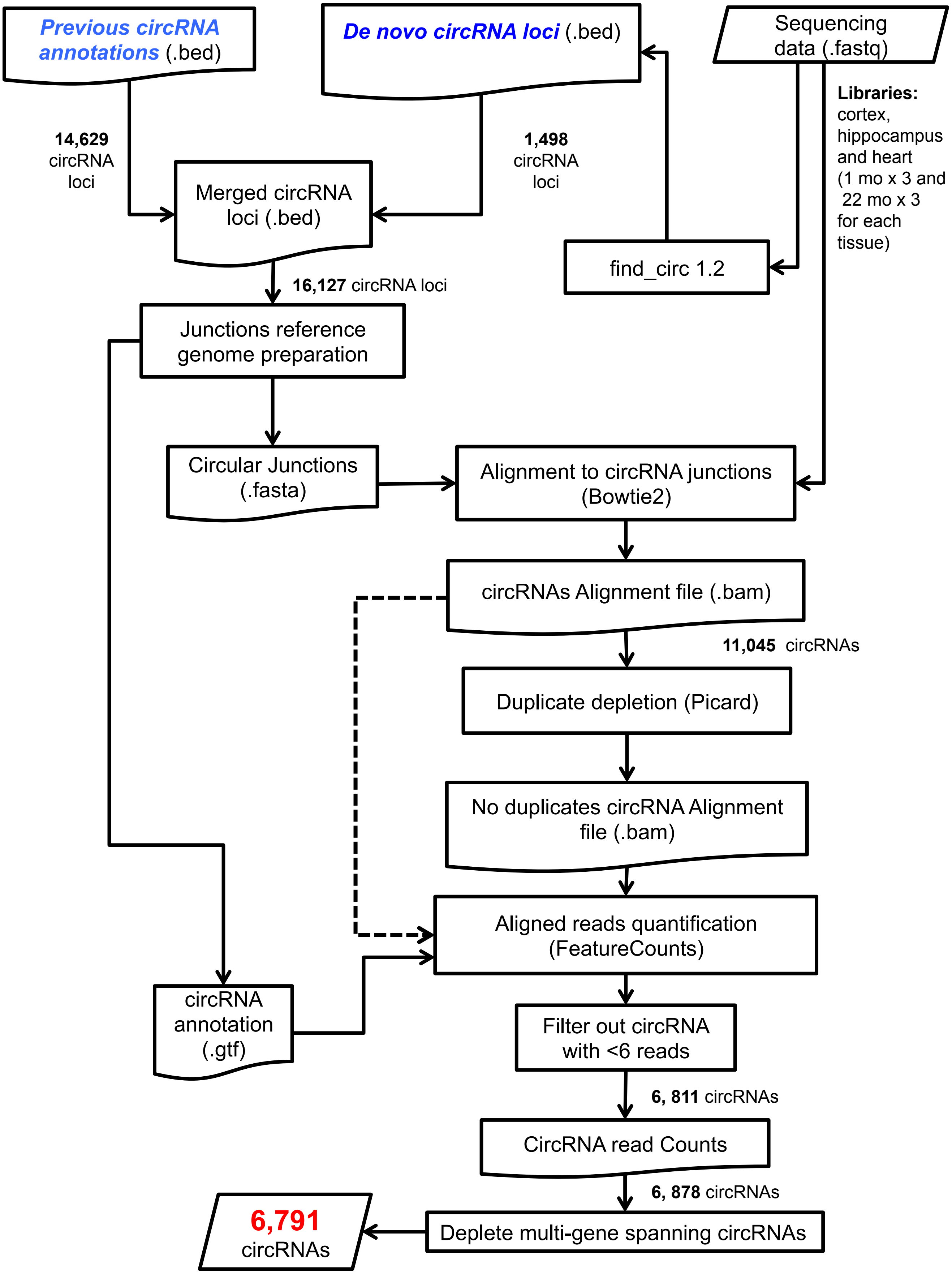

Supplementary Figure S1: Pipeline for circRNA annotation

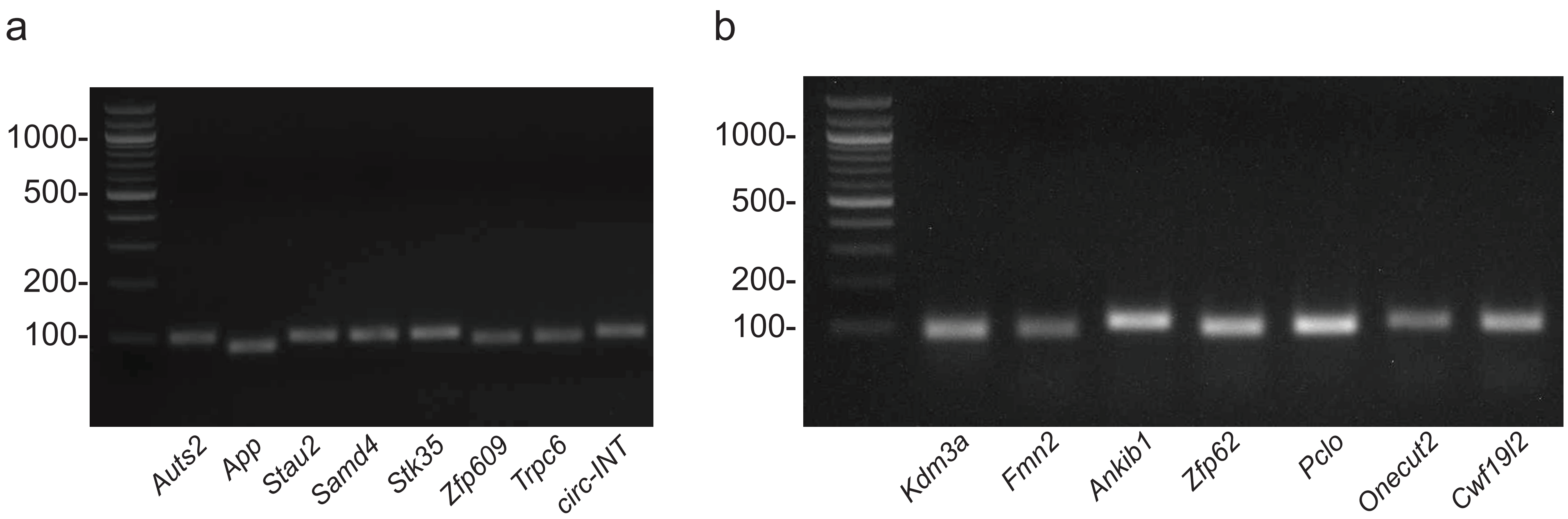

Supplementary Figure S2: PCR products from RT-qPCR reactions used to detect circRNAs

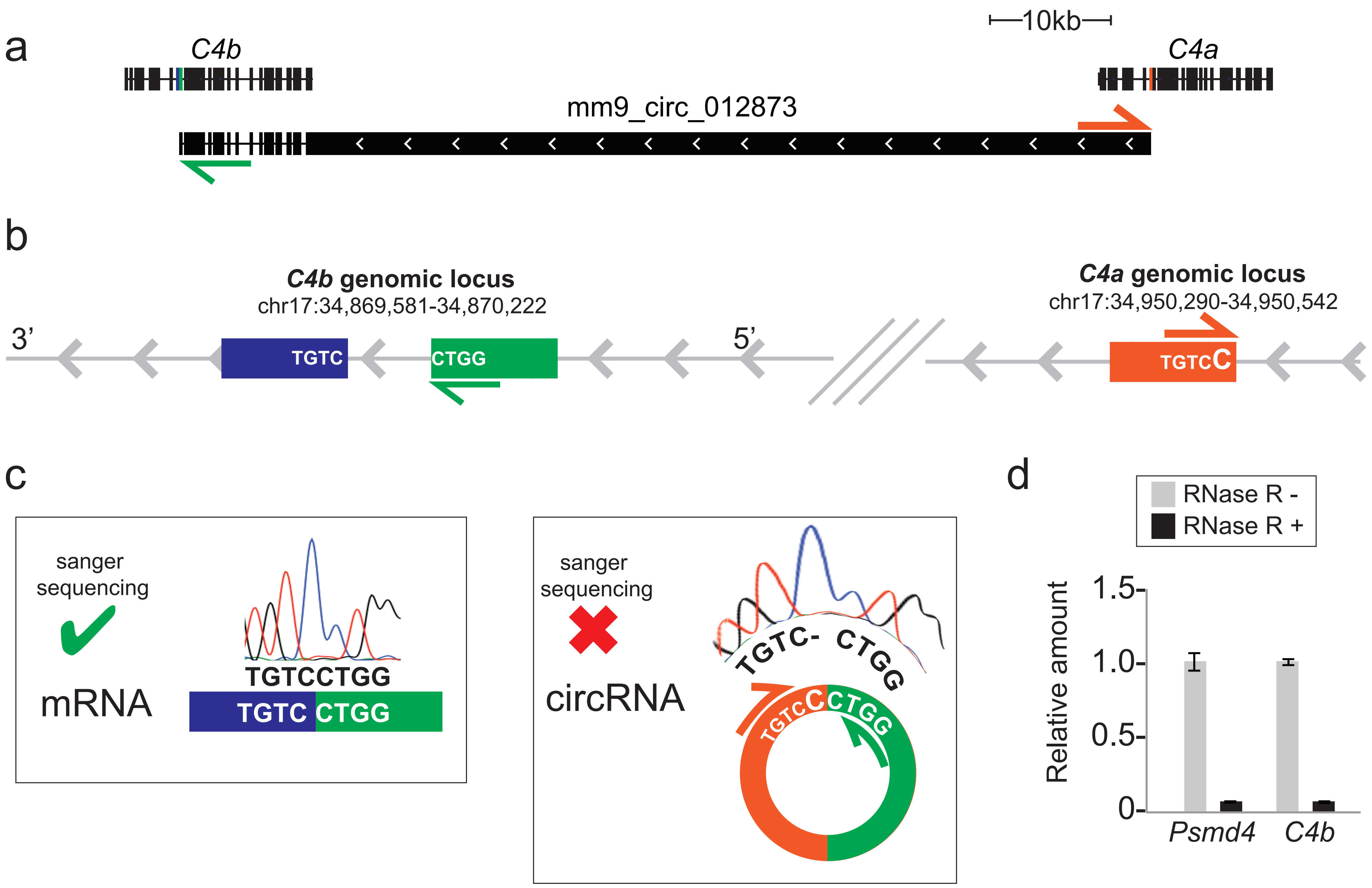

Supplementary Figure S3: Multi-gene spanning circRNAs are likely artifacts.

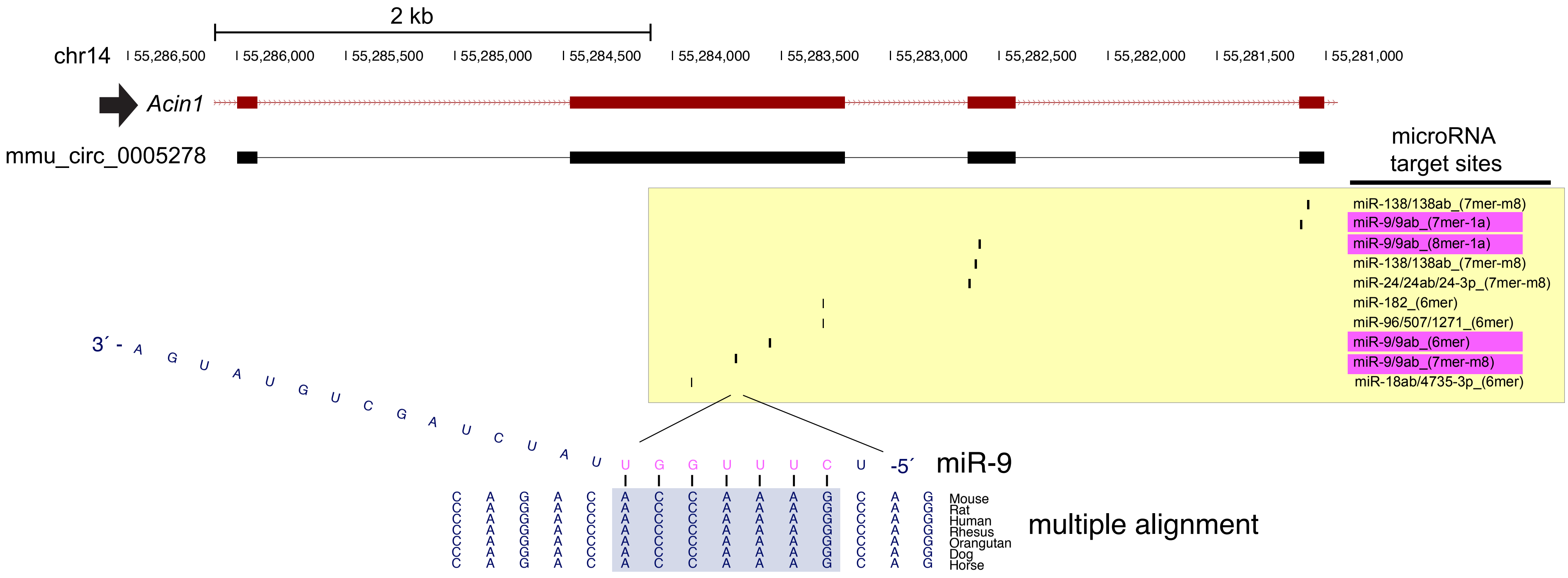

Supplementary Figure S4: MicroRNA seed sites in *Acin1* circRNA (mmu_circ_0005278)

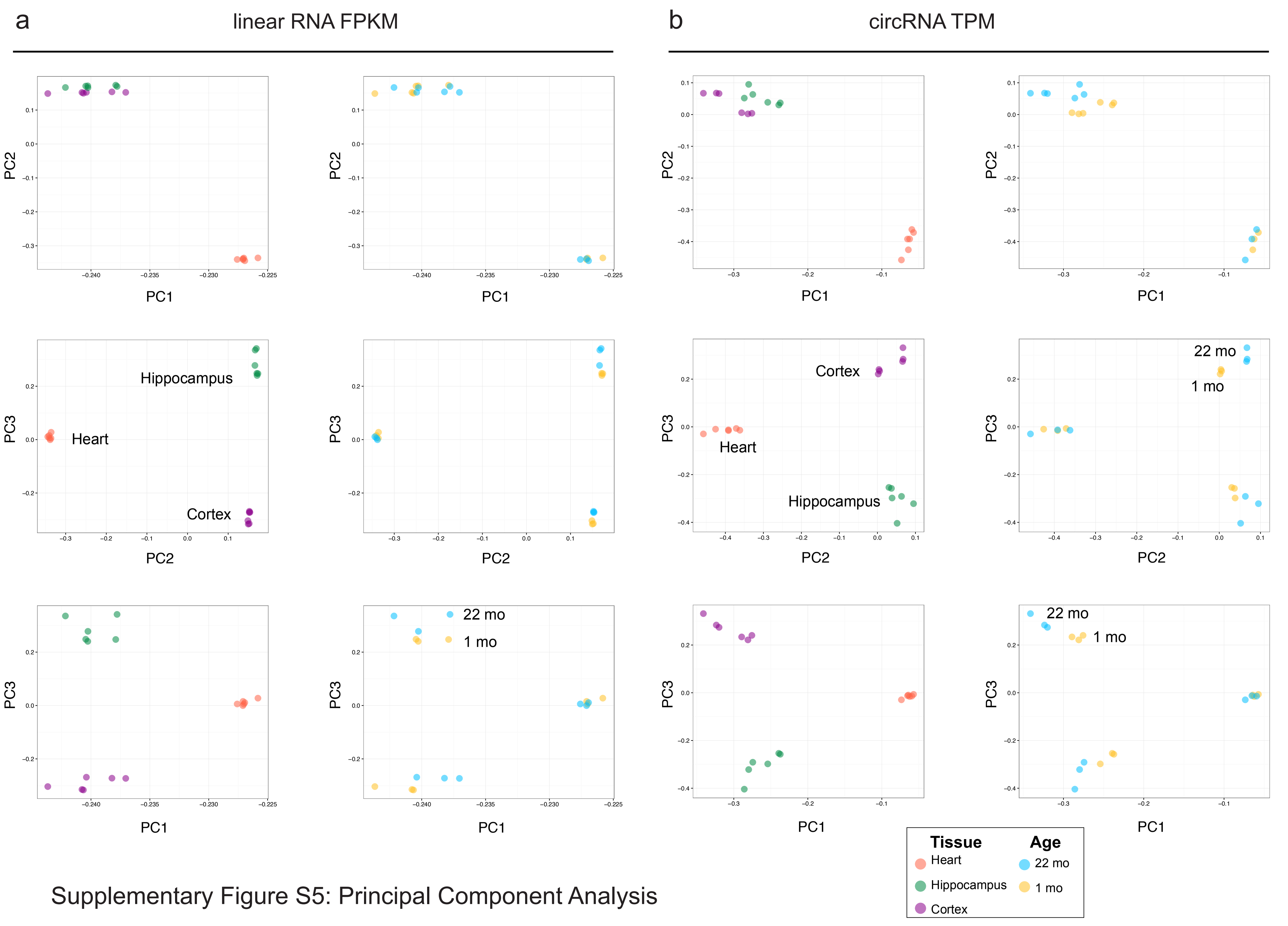

Supplementary Figure S5: Principal Component Analysis
